# Changes in species abundance after seven years of elevated atmospheric CO_2_ and warming in a Subarctic birch forest understorey, as modified by rodent and moth outbreaks

**DOI:** 10.7717/peerj.4843

**Published:** 2018-05-29

**Authors:** Brita M. Svensson, Bengt Å. Carlsson, Jerry M. Melillo

**Affiliations:** 1 Plant Ecology and Evolution, Department of Ecology and Genetics, Uppsala University, Uppsala, Sweden; 2 The Ecosystems Center, Marine Biological Laboratory, Woods Hole, MA, USA

**Keywords:** *Clethrionomys rufocanus*, *Epirrita autumnata*, Herbivory, Open-top chambers, Point-frequency analysis, *Vaccinium myrtillus*, *Vaccinium vitis-idaea*

## Abstract

A seven-year long, two-factorial experiment using elevated temperatures (5 °C) and CO_2_ (concentration doubled compared to ambient conditions) designed to test the effects of global climate change on plant community composition was set up in a Subarctic ecosystem in northernmost Sweden. Using point-frequency analyses in permanent plots, an increased abundance of the deciduous *Vaccinium myrtillus*, the evergreens *V. vitis-idaea* and *Empetrum nigrum* ssp. *hermaphroditum* and the grass *Avenella flexuosa* was found in plots with elevated temperatures. We also observed a possibly transient community shift in the warmed plots, from the vegetation being dominated by the deciduous *V. myrtillus* to the evergreen *V. vitis-idaea*. This happened as a combined effect of *V. myrtillus* being heavily grazed during two events of herbivore attack—one vole outbreak (*Clethrionomys rufocanus*) followed by a more severe moth (*Epirrita autumnata*) outbreak that lasted for two growing seasons—producing a window of opportunity for *V. vitis-idaea* to utilize the extra light available as the abundance of *V. myrtillus* decreased, while at the same time benefitting from the increased growth in the warmed plots*.* Even though the effect of the herbivore attacks did not differ between treatments they may have obscured any additional treatment effects. This long-term study highlights that also the effects of stochastic herbivory events need to be accounted for when predicting future plant community changes.

## Introduction

Over the past 135 years, the mean global temperature has increased by 0.85 °C and the atmospheric CO_2_ concentration has increased by about 120 ppmv. Climatic changes associated with continued increases in atmospheric CO_2_ are projected to be particularly marked in the boreal-tundra Arctic system (IPCC, 2014, p. 16) and could have large effects on the structure and function of these ecosystems. Tundra ecosystems contain huge stores of carbon in soil organic matter (Jones et al., 2014), and any shifts in the balance between photosynthesis and respiration could potentially have a major impact on carbon sequestration (Oechel et al., 1994).

The rise in high-latitude temperatures coincide with increased productivity, measured as ‘greenness’ using satellite observations (Myers-Smith et al., 2011). The increased greenness is proposed to partly be due to an increased shrub cover (Myers-Smith et al., 2011). This is supported by experimental research done over the last 20 years. Tundra ecosystems show a general increase in vegetation stature and in the cover of shrubs and graminoids and a decrease in the cover of mosses and lichens (Hollister, Webber & Tweedie, 2005). Many of the responses are site-specific as well as species-specific.

In a Scandinavian warming experiment, plant biomass increased, but because the studied species did not respond similarly, a shift in community composition also occurred (Kaarlejärvi et al., 2012). Such vegetation shifts have been recorded elsewhere (Kullman, 2002; Tape, Sturm & Racine, 2006; Tømmervik et al., 2009; Elmendorf et al., 2012; Gottfried et al., 2012) and the notion that higher temperatures could drive these changes are supported by warming experiments (Walker et al., 2006; Kaarlejärvi et al., 2012). In northern Sweden, vegetation changes consisting of increased shrub and tree density have been detected (Callaghan et al., 2011). One hypothesis is that these changes are caused by the changes in climate including growing-season length that has increased from 12.1 to 14.6 weeks in just under 30 years (Andrews et al., 2011). Manipulative experiments in Arctic tundra found that elevated temperature increased the abundance of graminoids and deciduous shrubs (Arft et al., 1999; Weintraub & Schimel, 2005; Walker et al., 2006; Elmendorf et al., 2012). For example, elevated temperatures increased the abundance of *Vaccinium myrtillus* at a site close to ours (Hartley et al., 1999).

At the same time that plant growth is stimulated by warming, carbon will be released as both plant and soil respiration increase (Biasi et al., 2008). Several studies have shown that in response to warming, Arctic ecosystems change from being net sinks to net sources of carbon to the atmosphere (Oechel et al., 1994; Mack et al., 2004; Biasi et al., 2008; Tarnocai et al., 2009). Arctic ecosystems are likely nitrogen limited (Chapin, 2003), and deciduous species should better be able to utilize the additional nitrogen that is projected to be released from soil organic matter under increased temperatures (Hartley et al., 1999). Indeed, a meta-analysis using data from two areas (Alaska and northern Sweden) showed that above-ground biomass, and particularly the biomass of deciduous and graminoid species, responded most strongly to nutrient addition (Van Wijk et al., 2003). The effects may, however, be of a transient nature (Alatalo & Little, 2014). Taken together we, therefore, hypothesize that warming, apart from the direct effect, will trigger a chain of events starting with increased soil organic matter decay leading to increased levels of available soil N, that, in turn, will stimulate plant growth. The addition of CO_2_ in combination with warming will likely further stimulate plant growth, while the addition of CO_2_ without warming will not stimulate plant growth because of nitrogen limitation.

Stochastic events may abruptly change the prerequisites for ecosystem function and make communities respond less predictably to changes in resource levels, for example biomass may vary dramatically in a non-linear fashion (Elmendorf et al., 2012; Olofsson, Te Beest & Ericsson, 2013). In Arctic and alpine areas, examples of stochasticity are topoclimatic events such as sudden late frosts in spring (Tenow & Nilssen, 1990) and outbreaks of herbivory (Bylund, 1997; Olofsson, Tømmervik & Callaghan, 2009; Olofsson, Te Beest & Ericsson, 2013). As deciduous species are more palatable to herbivores (Cornelissen et al., 2009; Dahlgren et al., 2009) we may expect species-specific responses. For example, the deciduous *V. myrtillus*, an important food resource for rodents (Bjärvall & Ullström, 1995; pers. obs.), particularly during winter (Dahlgren et al., 2007; Dahlgren et al., 2009; Soininen et al., 2013), reacted to simulated herbivory by producing new shoots, which the evergreen *V. vitis-idaea* did to a lesser degree (Tolvanen & Laine, 1997). In a study combining the effects of warming (which stimulated deciduous species’ growth) and simulated herbivory on *V. myrtillus* it was shown that this had a positive effect on evergreen species’ growth (Ylänne, Stark & Tolvanen, 2015), indicating a possible shift from deciduous to evergreen dwarf shrub species dominating the community.

Experimental investigations concerning the effects of global climate change have often been made over relatively short time periods (≤three seasons), not reflecting potential long-term changes in the ecosystem, but there are exceptions, e.g., (Rinnan et al., 2007; Kudo et al., 2010; Hill & Henry, 2011; Olofsson, Te Beest & Ericsson, 2013; Post, 2013; Ylänne, Stark & Tolvanen, 2015; Little et al., 2017). Long-term studies are important as responses may be transient due to a short-term peak in productivity in the early phases of a warming experiment (Seastedt & Knapp, 1993), or include a lag phase not picked up by short-term experiments (Arft et al., 1999).

The goals of our study were to investigate how long-term enhanced atmospheric CO_2_ concentrations and warming, singly and in combination, affect the ericaceous dwarf shrub-dominated Subarctic birch forest understorey in northern Sweden. However, during the seven-year study period (2000–2006) there was one peak (2001) in vole (*Clethrionomys rufocanus* Sundevall 1846) abundance (Olofsson, Tømmervik & Callaghan, 2012) and one (more severe, 2003 and 2004) of the Autumnal moth (*Epirrita autumnata* (Borkhausen, 1794)) (Babst, Esper & Parlow, 2010). These gave us a unique opportunity to analyse also the effects of environmental stochasticity on vegetation structure. Vole and moth outbreaks happen in a semi-regular pattern (Olofsson, Tømmervik & Callaghan, 2009; Olofsson, Te Beest & Ericsson, 2013). Depending on the strength of the outbreak (i.e., herbivore population size), the vegetation gets more or less severely damaged, especially in the case of the Autumnal moth: from complete removal of leaves from both trees and the field-layer flora to just smaller patches being hit (Karlsson et al., 2004).

In this paper we concentrate on the responses at the species and community levels, investigated using point-frequency analysis.

Based on the discussion above, we hypothesize that

(1) in warmed plots we will find higher abundance of vascular vegetation, particularly of deciduous dwarf shrubs and possibly also grasses,
(2) warmed plots would, accordingly, be more prone to herbivore attacks than non-warmed plots, and
(3) increased CO_2_ alone will not have any impact on vegetation; in combination with warming, CO_2_ levels will affect vegetation structure, as in point (1) above.

## Materials and Methods

### Study site

The study took place in a low-statured birch forest 10 km east of Abisko Scientific Research Station in the Subarctic region of Sweden (68°21′N, 19°4′E, 341 m a.s.l.). As is typical for the heath forest types of the region (Carlsson, Karlsson & Svensson, 1999), mountain birches (*Betula pubescens* ssp. *czerepanovii* (Orlova) Hämet-Ahti) were sparsely distributed and the field layer dominated by the three ericaceous dwarf shrubs *Vaccinium myrtillus* L., *V. vitis-idaea* L., and *Empetrum nigrum* ssp. *hermaphroditum* (Hagerup) Böcher. In smaller amounts, the dwarf shrub *V. uliginosum* L., the stoloniferous *Linnaea borealis* L., the herbaceous pteridophyte *Equisetum sylvaticum* L., and the grasses *Avenella flexuosa* (L.) Drejer and *Calamagrostis purpurea* (Trin.) Trin. were found. Common bryophytes were *Hylocomium splendens* (Hedw.) B. S. & G., *Dicranum* spp. (mainly *D. scoparium* Hedw. and *D. fuscescens* Turn.), *Pleurozium schreberi* (Brid.) Mitt. and species of *Barbilophozia* Loeske, while lichens were rare. The area was chosen as to ensure as little variation as possible within the site. The climate at the site is similar to that recorded at the Abisko Scientific Research Station, where the mean annual temperature is –0.5 °C and mean annual precipitation is 320 mm (Eriksson, 1982; Eriksson, 1983). The field experiment was approved by the County Administrative Board in Norrbotten, Sweden (no. 231-6447-01; 2502 035).

### Experimental setup

The experiment was established early in the growing season of 2000 and included a warming treatment and an elevated CO_2_ treatment in a blocked two-way factorial design, and was run until September 2006, i.e., for seven seasons. We used six c. 45-m^2^ blocks; within each block five experimental plots were established. Each plot was 1.5  × 0.75 m, and four of the five plots in each block were during the growing season (late May to early September) surrounded by a hexagonal open-top chamber (OTC) with a footprint of c. 1.5 m^2^. The walls of the OTC were made of non-UV-proof transparent acrylic sheets.

Four chambers in each of the six experimental blocks were randomly assigned to one of the following four treatments: (1) warmed chambers in which soil and air temperatures were elevated 5 °C above ambient, (2) CO_2_ chambers with doubled internal atmospheric CO_2_ concentrations, (3) combined warming and CO_2_-enrichment, (4) ambient control chambers receiving no elevated CO_2_ or warming. In addition, one plot, (5) “non-chambered control”, was established without the OTC in each of the six blocks, to discriminate treatment effects from experimental artefacts. Wooden boardwalks were erected to enable plots to be accessed without disturbing the vegetation.

The soil was warmed using resistance cables threaded through the organic upper layer of the soil. Heating cables were controlled by data loggers coupled to thermistors—three in warmed chambers, two in ambient chambers. The electricity through the cables was switched on and off automatically on a two-minute cycle to maintain the temperature difference between warmed and ambient chambers. Within the warmed chambers, three infrared lamps, suspended 1.2 m above the soil surface, warmed the plant surfaces with 22 Wm^−2^.

In CO_2_-enriched chambers, atmospheric concentrations were elevated to 730 ± 25 ppm CO_2_. A LiCor 6262 Infrared Gas Analyser monitored CO_2_ concentrations in four of the six enriched chambers. A data logger coupled to flow sensors and a mass flow controller regulated the flow of CO_2_ from the tanks to the chambers. CO_2_-enriched air was blown into chambers from two sides to maintain an even concentration of CO_2_ across each chamber. Non-CO_2_-treatment chambers had an identical design, but received air without CO_2_. This ensured that all chambers experienced similar effects of blowing, e.g., reduced convective heating effects within the chambers. Both warming and CO_2_-enrichment were applied during the growing season each year. The OTCs were removed during winter.

We set up one 0.75 × 0.75-m permanent subplot in each of the five treatments—warming, CO_2_, warming plus CO_2_, chambered, and non-chambered controls—within each block for vegetation and demography analyses. Homogeneity of vegetation was emphasized in the selection of the subplots to ensure as little variation as possible within each subplot.

### Point-frequency analysis

The occurrence of all vascular plants, bryophytes and lichens, as well as litter and bare soil, was recorded using a 10-mm-thick quadratic sheet of transparent acrylic glass. The sheet had 97 holes evenly drilled at 5-cm intervals and was standing on three adjustable metal legs enabling us to position the frame horizontally and in the same place every year, with the help of small metal markers on the soil surface and a water-level. The holes had been precision-drilled using a milling cutter to enable a 5-mm-diameter brass rod to be inserted with a minimum of angular error. All hits on a 20-mm-long zone on the sharpened tip of the rod were recorded as it was moved downward through the vegetation canopy. Whenever possible the final record would be that of litter (one or two hits) or a bryophyte. In the case of bryophytes, most of them might have more than one hit while *Barbilophozia* sp. never had more than one.

### Statistical analyses

Total number of point hits (a proxy for species abundance or dominance in 3D-space) for vegetation collectively (excluding litter), and for *V. myrtillus*, *V. vitis-idaea*, *E. nigrum* ssp*. hermaphroditum*, *A. flexuosa*, bryophytes and forbs, were analysed with Mixed effects ANOVAs with the random factor Block, and the fixed factors Year, Warming and CO_2_, the last two with two levels, and their interactions (Zar, 1999). To eliminate differences due to initial conditions the analyses were made using number of hits in 2000 as covariables. All data was log-transformed (*x*′ = ln(*x* + 1) before analyses to meet the assumptions of the statistical tests.

**Figure 1:**
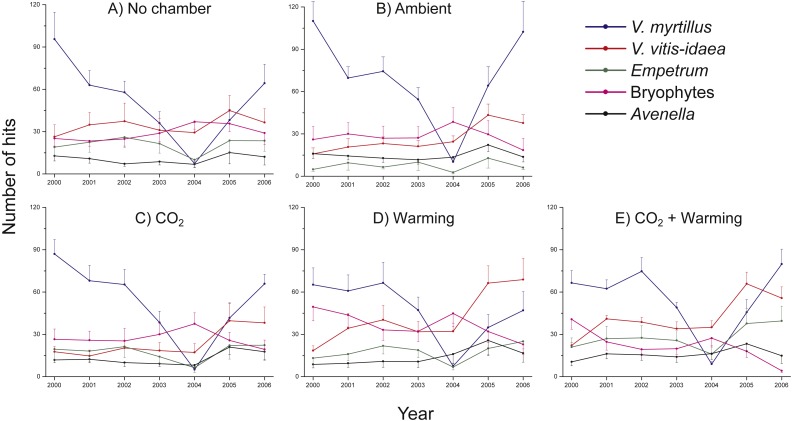
Species abundance in non-chambered control plots, and in the four treatment combinations: Ambient, CO_2_, Warming, and the combined treatment CO_2_ + Warming. Total number of hits for *Vaccinium myrtillus* (blue), *V. vitis-idaea* (red), *Empetrum nigrum* ssp. *hermaphroditum* (green), bryophytes collectively (purple), and *Avenella flexuosa* (black). The panels show (A) non-chambered control; B) Ambient; (C) CO_2_; (D) Warming; and (E) the combined treatment CO_2_ + Warming. Values are means (+ or –1 S.E.), *N* = 6.

There were no significant differences in number of hits between non-chambered controls and treatment controls (ambient) for any of the species studied, except *V. myrtillus* (Two-way ANOVA, Chamber: *F*_1,5_ = 4.8, *P* = 0.033; Year: *F*_1,5_ = 40.7, *P* < 0.001; Interaction Chamber × Year: *F*_1,5_ = 0.878, *P* = 0.502) and *A. flexuosa* (Two-way ANOVA, Chamber: *F*_1,5_ = 12.2, *P* = 0.001; Year: *F*_1,5_ = 3.13, *P* = 0.015; Interaction Chamber × Year: *F*_1,5_ = 0.832, *P* = 0.533). For both species there were initially more hits in the chambered control plot (*V. myrtillus*: 15%, *A. flexuosa*: 25%) (Fig. 1), but as the initial abundances of the species were included as co-variables in the analyses the results suggest that the chamber indeed had a positive influence, albeit small. However, since no other species showed any increased (or decreased) growth in the chambered control compared to the non-chambered control we henceforth did all analyses excluding the non-chambered controls to keep a balanced design.

As a proxy for canopy cover or dominance in 2D-space we analysed number of first hits per species in the same manner as for total number of hits.

We also tested whether there were any preferences by the voles and moths for any particular treatment. We did this by noting the degree of defoliation and the frequency of dead shoots on initially five marked *V. myrtillus* in each plot 2001–2006. In all cases we used a six-level scale, where 0 means no herbivory effects were detected. As above, a Mixed effects ANOVA with the random factor Block, and the fixed factors Year, Warming and CO_2_, the last two with two levels, and their interactions were used to test for differences between treatments. We also checked whether there were any differences in herbivory between chambered and non-chambered controls using a Two-way ANOVA with the fixed factors Year and Chamber and their interaction.

All analyses were made using IBM SPSS Statistics 24.0.0.0 (SPSS, Inc., Chicago, IL, USA).

## Results

The vegetation in the plots consisted of a typical birch heath forest field layer with *Vaccinium myrtillus*, *V. vitis-idaea*, *Empetrum nigrum* ssp*. hermaphroditum, Avenella flexuosa* and *Linnaea borealis* being present in all plots, the two former being the most common. The dominant bryophyte was *Hylocomium splendens*.

### Plant community responses to herbivory

Irrespective of treatment, there were marked differences between years, particularly regarding the abundance of *V. myrtillus* (Fig. 1A), with a small decrease in 2001, a recovery in 2002 followed by striking decreases in both 2003 and 2004. The decrease in vegetation abundance in the early phase of the experiment was caused by grazing by the Grey red-backed vole (*Clethrionomys rufocanus*) during the rodent peak in 2001. Data from separate demographic analyses (B Svensson, pers. obs., 2001)—where we followed the fates of individual shoots—show that 71 out of 183 (39%) *V. myrtillus* shoots were partly or completely grazed by rodents in 2001. The year after, 2002, we noted that shoots of *V. myrtillus* were still dying as a result of the herbivore attacks. In 2003, a heavy outbreak of the Autumnal moth (*Epirrita autumnata*) began in the area, including also our study site. The impact was even more severe in 2004 (Fig. 1A). For example, we observed that in most plots the shoots of *V. myrtillus* were completely defoliated.

**Figure 2:**
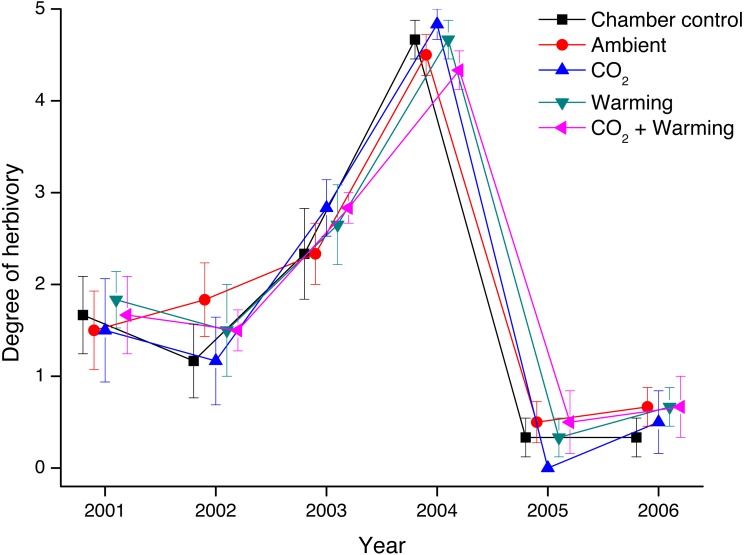
Degree of herbivory on *Vaccinium myrtillus* in non-chambered control plots, and in the four treatment combinations (Ambient, CO_2_, Warming, and the combined treatment CO_2_ + Warming) during the years 2001 through 2006. Effects of added CO_2_, warming and chamber on the degree of herbivory by *Clethrionomys rufocanus* and *Epirrita autumnata* on *Vaccinium myrtillus*, measured as survival and the degree of defoliation on marked shoots (five per treatment combination). Statistical analyses are presented in Table 1. Values are treatment means (±1 S.E.), *N* = 6.

Thus, the degree of herbivory on *V. myrtillus* differed markedly between years (Fig. 2, Table 1). There was, however, no difference in the degree of herbivory between treatments (Mixed ANOVA, Table 1). Also, there was no difference between chambered and non-chambered controls (*F*_1,60_ = 0.527, *P* = 0.471).

**Table 1:** Effects of added CO_2_ and warming on the degree of herbivory on *Vaccinium myrtillus*. Effects of added CO_2_ and warming on the degree of herbivory by *Clethrionomys rufocanus* and *Epirrita autumnata* on *Vaccinium myrtillus*, measured as survival and the degree of defoliation on marked shoots (five per treatment combination). Data are shown in Fig. 2.

	*df*	*F*	*P*
Warming	1, 177	0.862	0.354
CO_2_	1, 177	0.024	0.877
**Year**	**6, 177**	**127**	**<0.001**
Warming × CO_2_	1, 177	0.002	0.965
Warming × Year	6, 177	0.289	0.942
CO_2_× Year	6, 177	0.360	0.903
Warming × CO_2_× Year	6, 177	0.461	0.836

**Notes.**

Bolded text indicate significance below *P* = 0.05.

### Plant community responses to warming and CO_2_ enrichment

**Figure 3:**
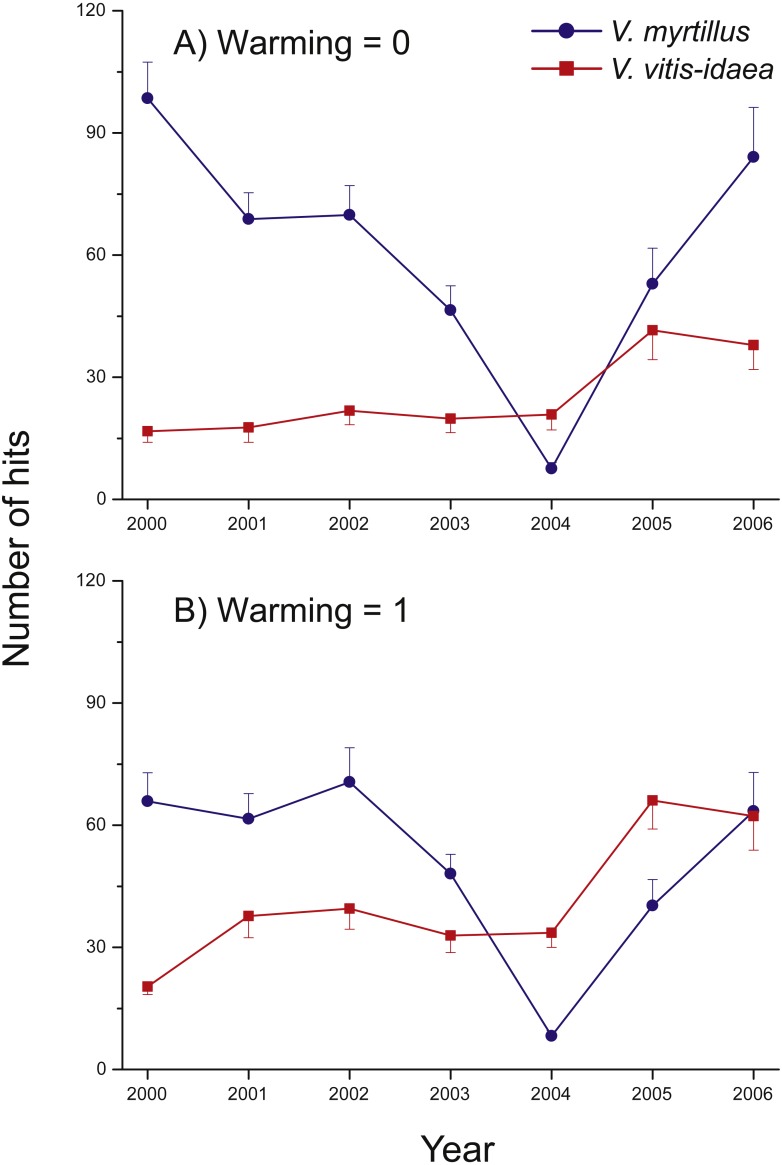
Species abundance in the two un-warmed and the two warmed treatment combinations during the years 2000 through 2006 for *Vaccinium myrtillus* (blue) and *V. vitis-idaea* (red). Total number of hits in the two un-warmed (A) and the two warmed (B) treatment combinations during the years 2000 through 2006 for *Vaccinium myrtillus* (blue) and *V. vitis-idaea* (red). Values are means (+ or −1 S.E.), *N* = 12.

A notable change was detected in the warmed plots in 2004 and 2005, where we found a shift in community composition (measured as the total number of hits), from the deciduous *V. myrtillus* to the evergreen *V. vitis-idaea* becoming the most common species (Fig. 3). In 2006, the two species were equally common so the shift may have been of a transient nature.

**Figure 4:**
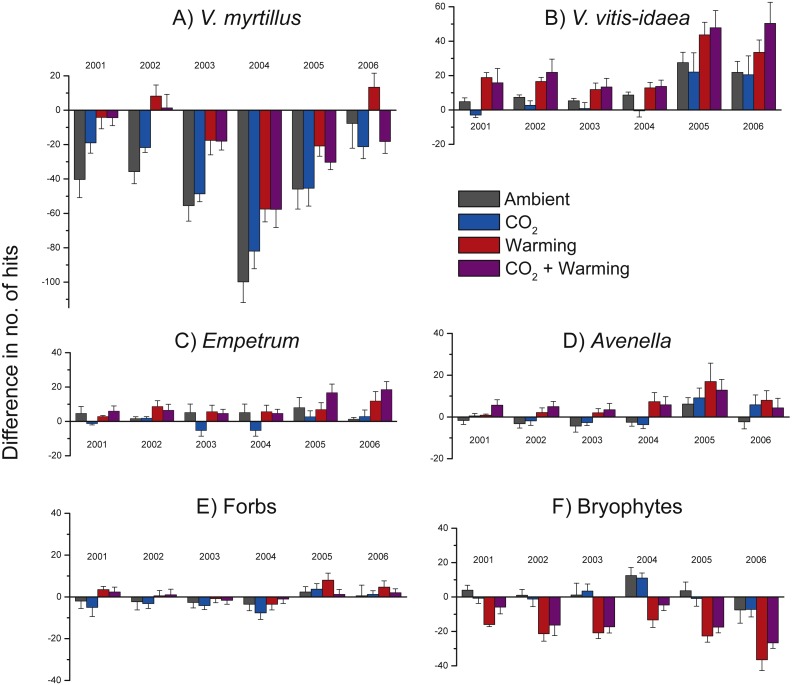
Change in species abundance in the years 2001 through 2006 when compared to the abundance in 2000 for the most common species and in the four treatment combinations. Difference between the number of hits in 2001 through 2006 and the number of hits in 2000 (at the start of the experiment) for (A) *Vaccinium myrtillus*; (B) *V. vitis-idaea*; (C) *Empetrum nigrum* ssp. *hermaphroditum*; (D) *Avenella flexuosa*; (E) forbs collectively; and (F) bryophytes collectively. Positive values indicate an increase in abundance while negative values indicate a decrease in abundance. Statistical analyses are presented in Table 2. Values are treatment means (+ or −1 S.E.), *N* = 6.

Because of initial differences between chambers we illustrate treatment effects using differences between each year and the *starting* year (i.e., no. of hits in 2001 minus no. of hits in 2000, no. of hits in 2002 minus no. of hits in 2000, etc. (Fig. 4). Positive effects of warming were found for *V. myrtillus*, *V. vitis-idaea*, *E. nigrum* ssp. *hermaphroditum*, *A. flexuosa*, and a near to significant positive effect also on forbs collectively (Fig. 4, Table 2). Although there was a strong general decrease in the number of hits for *V. myrtillus* (due to herbivory), *V. myrtillus* in the warmed plots fared relatively better (Fig. 4A, Table 2b). For *V. myrtillus* we also found a significant three-way interaction effect Warming × CO_2_ × Year (Table 2b), probably due to the years 2002 and 2006 where warming alone had a more positive effect than combined with elevated CO_2_, as opposed to the other years (Fig. 4A). Not until the last year (2006) were the abundance values back to the same level as before the vole and moth outbreaks, and only so in the warmed plots. One of the blocks had fewer point hits initially and also reacted differently to vole grazing. This was due to the lower abundance of *V. myrtillus* in this block.

**Table 2:** Effects of six years of added CO_2_ and warming on species abundances. Effects of six years of added CO_2_ and warming on the abundance of (a) total vegetation cover; (b) *Vaccinium myrtillus*; (c) *V. vitis-idaea*; (d) *Empetrum nigrum* ssp. *hermaphroditum*; (e) *Avenella flexuosa*; (f) forbs collectively; and (g) bryophytes collectively, measured as the total number of point hits in the years 2001 to 2006, using number of hits in 2000 as covariable. Data are shown in Fig. 4.

	*Df*	*F*	*P*
**(a) Total vegetation**			
**Warming**	**1, 118**	**50.7**	<**0.001**
CO_2_	1, 114	3.05	0.084
**Year**	**5, 114**	**58.1**	<**0.001**
Warming × CO_2_	1, 115	2.64	0.107
Warming × Year	5, 114	0.810	0.545
CO_2_ × Year	5, 114	0.663	0.652
Warming × CO_2_ × Year	5, 114	0.325	0.897
**Total vegetation 2000 (covariable)**	**1, 33.6**	**11.3**	**0.002**
**(b) *V. myrtillus***			
**Warming**	**1, 94.9**	**11.6**	**0.001**
CO_2_	1, 116	1.20	0.275
**Year**	**5, 115**	**86.7**	<**0.001**
**Warming × CO_2_**	**1, 118**	**7.00**	**0.009**
Warming × Year	5, 115	1.89	0.101
CO_2_ × Year	5, 115	1.20	0.312
Warming × CO_2_ × Year	5, 115	1.60	0.166
***V. myrtillus* 2000 (covariable)**	**1, 26.5**	**69.8**	**< 0.001**
**(c) *V. vitis-idaea***			
**Warming**	**1, 115**	**12.9**	<**0.001**
**CO_2_**	**1, 116**	**22.0**	<**0.001**
**Year**	**5, 114**	**16.1**	<**0.001**
**Warming × CO_2_**	**1, 114**	**8.64**	<**0.004**
Warming × Year	5, 114	0.358	0.876
CO_2_ × Year	5, 114	0.539	0.747
Warming × CO_2_ × Year	5, 114	0.525	0.757
***V. vitis-idaea* 2000 (covariable)**	**1, 116**	**146**	<**0.001**
**(d) *Empetrum***			
**Warming**	**1, 114**	**28.1**	<**0.001**
CO_2_	1,****116	1.752	0.188
**Year**	**5, 114**	**11.0**	<**0.001**
**Warming × CO_2_**	**1, 114**	**5.66**	**0.019**
Warming × Year	5, 114	0.549	0.739
CO_2_ × Year	5, 114	0.127	0.986
Warming × CO_2_ × Year	5, 114	0.554	0.735
***Emperum* 2000 (covariable)**	**1, 116**	**361**	<**0.001**
**(e) *Avenella***			
**Warming**	**1, 114**	**12.5**	**0.001**
CO_2_	1, 114	1.04	0.309
**Year**	**5, 114**	**6.23**	<**0.001**
Warming × CO_2_	1, 114	2.50	0.117
Warming × Year	5**,**114	0.456	0.808
CO_2_ × Year	5, 114	0.214	0.956
Warming × CO_2_ × Year	5, 114	1.08	0.376
***Avenella* 2000 (covariable)**	**1, 118**	**158**	<**0.001**
**(f) Forbs**			
Warming	1, 117	0.053	0.819
**CO_2_**	**1, 114**	**4.96**	**0.028**
**Year**	**5, 114**	**10.4**	<**0.001**
Warming × CO_2_	1, 114	0.001	0.978
Warming × Year	5, 114	0.546	0.741
CO_2_ × Year	5, 114	0.308	0.907
Warming × CO_2_ × Year	5, 114	1.400	0.230
**Forbs 2000 (covariable)**	**1, 117**	**22.4**	<**0.001**
**(g) Bryophytes**			
**Warming**	**1, 92.3**	**46.9**	<**0.001**
**CO_2_**	**1, 115**	**13.1**	<**0.001**
**Year**	**5, 115**	**14.8**	<**0.001**
Warming × CO_2_	1, 117	3.38	0.069
Warming × Year	5, 115	0.607	0.695
CO_2_ × Year	5, 115	0.549	0.739
Warming × CO_2_ × Year	5, 115	1.31	0.264
**Bryophytes 2000 (covariable)**	**1, 17.5**	**158**	<**0.001**

**Notes.**

Bolded text indicate significance below *P* = 0.05.

A statistically significant negative effect of added CO_2_ was found for the other common dwarf shrub, *V. vitis-idaea*, (Fig. 4B, Table 2c). This species did not react as negatively to the vole and moth outbreaks; its abundance never went below the starting level (Fig. 4B). In 2005 and 2006, *V. vitis-idaea* recovered from the herbivore attacks also in the other two treatments. In contrast to *V. myrtillus*, no grazing was observed on *V. vitis-idaea* leaves.

The dwarf shrub *Empetrum nigrum* ssp. *hermaphroditum* did not seemingly react as negatively to the vole or moth outbreaks as *V. myrtillus* but still had its lowest abundance values in 2004. *Empetrum* responded positively to warming as can be seen by the increased difference in number of hits in 2005 and 2006 in the two warmed treatment combinations (Fig. 4C, Table 2d). There was only one graminoid species in the analysed plots, viz. *Avenella flexuosa*, and this species also increased in the two warmed treatments (Fig. 4D, Table 2e). The other, less common herbaceous vascular plant species (i.e., forbs collectively) were unevenly distributed between the plots and only showed a significant response to the experimental treatments at the *P* < 0.1 level (Fig. 4E, Table 2f), nor could we see any herbivory effects.

In contrast to the dwarf shrubs, warming decreased the abundance of bryophytes (Fig. 4F, Table 2g), particularly in the last year. Also, bryophytes did not respond strongly to herbivory outbreaks as the notable decrease in the years with herbivore attacks for *V. myrtillus* is less marked for bryophytes (Fig. 4F). We also found a significant positive effect of added CO_2_, and a significant interaction effect between added CO_2_ and warming (Table 2g), as seen by the decrease in the number of hits in the combined treatment compared to the slightly positive effect in the CO_2_-only plots (Fig. 4F).

We also tested whether species composition of the top-most, first hit, differed between treatments, which would suggest a change in canopy cover between species. The significant positive effect of warming remained for the vegetation as a whole and for *V. vitis-idaea* and *E. nigrum* ssp. *hermaphroditum* but disappeared for *V. myrtillus* and *A. flexuosa*. The pattern that in 2004 and 2005 *V. myrtillus* became less common than *V. vitis-idaea* in the warmed plots remained, however. There was still a strong negative effect of warming on the abundance of bryophytes.

## Discussion

Our main findings were that warming increased the abundances of the ericaceous dwarf shrubs *V. myrtillus, V. vitis-idaea*, and *E. nigrum* ssp. *hermaphroditum*, and the grass *A. flexuosa* (Fig. 4), and that this led to a, possibly temporary, shift in community composition, from a dominance of the deciduous *V. myrtillus* to the evergreen *V. vitis-idaea* in the warmed plots (Fig. 3). We also found reduced abundance of *V. vitis-idaea* in CO_2_-enriched treatment plots (Fig. 4B), while bryophytes collectively showed increased abundance when CO_2_ was added and that warming decreased their abundances (Fig. 4F).

Furthermore, due to the sudden and unexpected outbreaks of, first, Grey-backed vole and later, during two seasons, Autumnal moth, we got the chance to monitor how the responses to altered environmental conditions were modified by these stochastic events (Olofsson, Te Beest & Ericsson, 2013).

### Accelerated growth in the warmed plots

The evergreen dwarf shrub *V. vitis-idaea* and the deciduous *V. myrtillus* were the most responsive species to the warming treatments (warmed and combined warming + CO_2_). The third ericaceous species, the evergreen *E. nigrum ssp. hermaphroditum* reacted similarly and positively to warming, but not as strongly. An increase in shrub abundance is as expected (Myers-Smith et al., 2011) and also in accordance with the meta-analysis results of Arft et al. (1999).

As hypothesized, *V. myrtillus* was the species responding the strongest to warming, as was also found by Van Wijk et al. (2003) in their meta-analysis of over 40 studies. There was also a small positive chamber effect on the growth of this species. However, the difference between treatments was not particularly large, probably because the response was obscured by the herbivore attacks. *Vaccinium myrtillus* was particularly liked by the Grey red-backed vole during its population peak in 2001 (pers. obs.), which explains the decrease in abundance of *V. myrtillus* in this year compared to the start of the experiment (2000). Judging from the strong negative response in *V. myrtillus* in the years 2003 and 2004, where the average number of hits per plot was reduced by 47 and 91% (Fig. 1), respectively, it seems very likely that this species was particularly palatable also to *E. autumnata* larvae.

In addition, herbivores may affect soil nutrient availability as a direct effect of faeces deposition. Since the herbivores may eat substantial amounts of the green biomass, an increase in available soil nutrients is expected from dying roots and from faeces, particularly from invertebrate faeces (frass), as these are readily decomposed (Kagata & Ohgusju, 2012) and can increase nutrient availability (Hollinger, 1986; Lightfoot & Whitford, 1990; Frost & Hunter, 2004; Frost & Hunter, 2007). For example, it has been suggested that frass excreted by *E. autumnata* larvae may fertilize *Betula pubescens* spp. *czerepanovii* (Haukioja, Suomela & Neuvonen, 1985), the overstorey species at our experimental site. This is not surprising as frass may contain nearly 90% of the leaf N that was originally consumed (Hollinger, 1986; Lightfoot & Whitford, 1990; Frost & Hunter, 2007). *Avenella flexuosa* was the only grass species present in the analysed plots and, as hypothesized, responded positively to warming. This fits well with the above as an increase in grasses is expected when nutrient availability is increased, which has been demonstrated in a number of studies (e.g., Karlsen et al., 2013), and in relation to global warming in a meta-analysis study by Van Wijk et al. (2003). We also detected an increase in *A. flexuosa* flowering frequencies in 2005 and 2006, which may be due to the increased light levels after the herbivore attacks (Scurfield, 1954). Due to the positive feedback loops emanating from the released nutrients from herbivores (carcasses and frass) and dead roots (Kaarlejärvi, Hoset & Olofsson, 2015) the vegetation will recover quickly, even in the absence of extra CO_2_ or warming. An additional nutrient source may be the litter from *E. nigrum* ssp. *hermaphroditum*. We observed that the cuticulas on young shoots of this species were gnawed by the moths, and litter from this species increased at the same time as living shoots increased. Interestingly, due to the high phenolic content in *E. nigrum* ssp. *hermaphroditum* leaves, its litter decomposes slowly and an organic top soil layer is formed. Nutrients are thus mainly available for plants with ericoid mycorrhiza (Tybirk et al., 2000 and references therein) and this may benefit the two *Vaccinium* species in our study.

We did not find the expected higher levels of herbivory in the warmed plots. If the effect of warming induces higher resilience from stochastic disturbances in subarctic systems by boosting plant growth after herbivore attacks, the effect of the herbivores may be obscured. Or is it the herbivores that contribute to the resilience of the system by intermittently removing biomass, returning the vegetation to its original lower level, as suggested by Kaarlejärvi, Hoset & Olofsson (2015)? Such top-down controls have been shown to be strengthened by warming in cold regions (Marino, Romero & Farjalla, 2018).

### A shift in community composition?

*Vaccinium vitis-idaea* strongly benefitted from the reduced presence of *V. myrtillus*, as it increased in all plots, particularly in the warmed plots. In particular, the abundance of taller shoots of *V. myrtillus* was reduced (as seen by the non-significant effect of warming on the number of first hits). This resulted in a community shift. Likewise, Rinnan, Stark & Tolvanen (2009) simulated *V. myrtillus* herbivory and found decreased abundance, which at the same time benefitted evergreen shrubs (Ylänne, Stark & Tolvanen, 2015). However, as the two species were again equally common in the last year of the experiment, the shift may be of a transient nature, perhaps because of the particularly positive effect of warming on *V. myrtillus*. The fact that *V. myrtillus* reacted positively (albeit weakly) on the presence of the chamber itself supports this.

Even though *V. vitis-idaea* is only occasionally used as a food source for most vole species (Kalela, 1957; Batzli & Lesieutre, 1991), an explanation for the positive response in the warmed plots could be an increased defence from secondary substances in the leaves of *V. vitis-idaea*. Concentrations of tannins have been suggested to increase after warming and nutrient addition (Graglia et al., 2001; Hansen et al., 2006). However, this does not seem likely in our case as the degree of herbivory did not differ between treatments. In addition, others have found little or no effects on tannin concentrations with warming (Kaarlejärvi et al., 2012). Interestingly, during our demographic analyses (B Svensson, pers. obs., 2003; 2004) we observed that shoot apices, rather than leaves, of *V. vitis-idaea* were consumed by the Autumnal moth, which in the following year was followed by a substantial increase in the number of side shoots. So, irrespective of a possible leaf tannin concentration increase, *V. vitis-idaea* increased in abundance due to released apical dominance resulting in increased branching (Svensson & Callaghan, 1988) as a response to herbivory.

### Bryophytes

Bryophytes reacted negatively to warming, which has been shown before (Healey, Oberbauer & Hollister, 2016). However, a low number of hits for bryophytes, particularly in combination with many hits for vascular plants, does not necessarily mean that bryophytes have decreased in frequency when the abundance of vascular plants increased. Rather, as the vegetation at this site is dense it may be an effect of bryophytes being less visible under the vascular plant layer and an artefact of the point-frequency analysis method. This is indicated by the increased number of bryophytes hits in the year 2004, when herbivory intensity was high and vascular plant abundance reduced. This increase could also, however, be a response to increased light levels this year.

### Minor effects of added CO_2_ alone

We did not expect any response to elevated CO_2_ alone, as tundra plants generally are nutrient limited. However, in an earlier study using the same experiment (Olsrud et al., 2010) where we evaluated the effects on global climate change on ericoid mycorrhizal function after two years’ exposure, we found an increased soil organic N availability in the CO_2_-only plots. This could probably be a response to mycorrhizal activity in CO_2_-plots (Emmerton et al., 2001; Read & Perez-Moreno, 2003) as a simultaneous increased mycorrhizal colonization was found under elevated CO_2_ (Olsrud et al., 2010). This might explain the transient positive response to CO_2_ found for *V. myrtillus* during the first two years of the experiment. The significant *negative* effect of added CO_2_ on *V. vitis-idaea* contradicts this. However, see Feng et al. (2015) for possible mechanisms whereby increased CO_2_ might constrain nitrogen acquisition and growth.

In many global climate change experiments, we find transient above-ground positive growth responses to enhanced CO_2_ (e.g., Oechel et al., 1994; Arft et al., 1999). For example, an increased growth of forest trees was seen initially after which it declined (Fig. 4 in Körner, 2006). This is because the plants enter a new steady-state regarding, e.g., leaf area and root turnover (Körner, 2006). However, herbivory may interact with how the vegetation is responding to these changes in CO_2_ concentration. A strong herbivore attack will offset this steady-state as a result both from released above-ground competition (as the canopy is more or less destroyed, depending on the size of the herbivore attack) as well as an increase in soil nutrients after the decomposition of dead roots and the direct fertilizing effect of herbivores in the form of frass. Vegetation responses to enhanced CO_2_ will start anew after the herbivore attack.

##  Supplemental Information

10.7717/peerj.4843/supp-1 Data S1 Data for seven years, one year per excel-file plus one excel-file with keys

## Additional Information and Declarations

### Field Study Permissions

The following information was supplied relating to field study approvals (i.e., approving body and any reference numbers): The field experiment was approved by the County Administrative Board in Norrbotten, Sweden (no. 231-6447-01; 2502 035).

### Data Availability

The following information was supplied regarding data availability: The raw data are provided in the Supplemental File.
